# Sex differences in a mouse model of diet-induced obesity: the role of the gut microbiome

**DOI:** 10.21203/rs.3.rs-3496738/v1

**Published:** 2023-11-01

**Authors:** Saluda Stapleton, Grace Welch, Lindsay DiBerardo, Linnea Ruth Freeman

**Affiliations:** Furman University; Furman University; LECOM: Lake Erie College of Osteopathic Medicine; Furman University

**Keywords:** sex differences, gut microbiome, metabolic profile, diet-induced obesity, short chain fatty acids

## Abstract

**Background:**

Recent decades have seen an exponential rise in global obesity prevalence, with rates nearly doubling in a span of forty years. A comprehensive knowledge base regarding the systemic effects of obesity is required to create new preventative and therapeutic agents effective at combating the current obesity epidemic. Previous studies of diet-induced obesity utilizing mouse models have demonstrated a difference in bodyweight gain by sex. In such studies, female mice gained significantly less weight than male mice when given the same high fat (HF) diet, indicating a resistance to diet-induced obesity. Research has also shown sex differences in gut microbiome composition between males and females, indicated to be in part a result of sex hormones. Understanding metabolic differences between sexes could assist in the development of new measures for obesity prevention and treatment. This study aimed to characterize sex differences in weight gain, plasma lipid profiles, fecal microbiota composition, and fecal short chain fatty acid levels. We hypothesized a role for the gut microbiome in these sex differences that would be normalized following microbiome depletion.

**Methods:**

A mouse model was used to study these effects. Mice were divided into treatment groups by sex, diet, and presence/absence of an antibiotic cocktail to deplete genera in the gut microbiome. We hypothesized that sex differences would be present both in bodyweight gain and systemic measures of obesity, including hormone and circulating free fatty acid levels.

**Results:**

We determined statistically significant differences for sex and/or treatment for the outcome measures. We confirm previous findings in which male mice gained significantly more weight than female mice fed the same high fat diet. However, sex differences persisted following antibiotic administration for microbiome depletion.

**Conclusions:**

We conclude that sex differences in the gut microbiome may contribute to sex differences in obesity, but they do not explain all of the differences.

## Summary

This study investigated how differences in metabolism between males and females could play a role in the development of new methods of obesity prevention and treatment. Two-month-old male and female mice were divided into a total of eight experimental groups based on sex, diet, and the presence or absence of antibiotic treatment, which was administered over a period of six weeks after 11 weeks of dietary treatment. At the end of 17 weeks, final body weights were recorded. Plasma and fecal samples were also collected to analyze differences in lipid metabolism, composition of gut bacteria, and levels of short chain fatty acids between the sexes. We suspected that the gut bacteria may have an impact on metabolism and nutrient absorption and that altering the composition through antibiotic treatment might normalize the effects. However, the results show that male mice on a high-fat diet gained more weight than female mice on the same diet with and without antibiotic treatment. We also observed a difference in the makeup of the gut microbiome between male and female mice, which emphasizes the impact that specific bacteria have on body weight, adiposity, and metabolism. In addition, we found sex differences in the levels of short chain fatty acids and plasma free fatty acids, but they are not yet well-characterized and more research is necessary. In conclusion, sex differences in the gut microbiome may contribute to sex differences in obesity, but they do not explain all the differences observed.

## Background

Obesity is an epidemic in our country, and continues to rise as a global problem. In 1975, the global obesity rate was 20.2%, affecting approximately 30.7 million men and 69.3 million women. In 2016, the global obesity rate had climbed to 39.1%, affecting approximately 281 million men and 390 million women (1). Recent data (2020) reports a prevalence of obesity in the United States to be 41.9%, with a prevalence percentage of 41.6% for men and 42.1% for women (2). Not only has obesity increased significantly throughout the world, it is important to note sex differences in obesity. These sex differences existed before obesity rates rose across the globe, as shown by the statistics above.

Studies in rodents and humans have shown that obesity can cause significant changes to the gut microbiota. Broadly, an increase in *Firmicutes* and a reduction in *Bacteroidetes* have been observed in a number of studies (3–5). This microbial phenotypic profile is thought, in part, to increase capacity for the fermentation of carbohydrates (6). High levels of carbohydrates as well as saturated fats are found in the “Western diet” (7). An alteration to the gut microbial community that leads to decreased microbial diversity and number of commensal bacteria is termed “dysbiosis”. Gut dysbiosis is associated with many different chronic health conditions including inflammatory bowel disease, metabolic syndrome, cardiovascular disease, obesity, and some cancers (8, 9). Sex differences in the gut microbiome have also been described. In a study that evaluated 89 different inbred strains of mice, with a total population of 689 mice, a number of taxa were revealed to be different between males and females when specific genetic backgrounds were evaluated, as genetic background can also affect the microbiota. This study (10) and others have implicated sex hormones as one of the factors that shape sex differences in the gut microbiota (10–12). For example, Yurkovetskiy et al. revealed sex differences in gut microbiota composition were dependent on sex hormones, not X-chromosome-associated factors, after measuring gut microbiome composition for male, female, and castrated male mice (13). Org et al. (2016) have also shown that hormones affect bile acid profiles, and it has previously been shown that bile acids affect the gut microbiota (14–16). Bile acid synthesis and bile acid pool sizes are typically higher in females compared to males (17).

The gut microbiome plays an important role in aiding digestion, metabolism, growth, development, and proper immune system functioning (18). More specifically, the gut microorganisms participate in nutrient absorption as well as synthesis of enzymes, vitamins, amino acids, and short chain fatty acids (SCFAs). SCFAs have 1–6 carbon atoms and are produced via bacterial fermentation of dietary fiber. The three main SCFAs produced by bacteria are butyrate, propionate, and acetate. Briefly, butyrate is the main energy source for colonocytes and can activate intestinal gluconeogenesis. Propionate is transferred to the liver where it regulates gluconeogenesis as well as satiety signaling. Acetate is the most abundant SCFA and it is essential for the growth of other intestinal bacteria. Acetate can also be transferred to peripheral tissues where it participates in cholesterol metabolism and lipogenesis (5). Previous studies have shown that higher production of SCFAs correlates with reduced diet-induced obesity and reduced insulin resistance. SCFAs activate fatty acid oxidation and inhibit de novo fatty acid synthesis and lipolysis (3).

Sex differences in obesity for the human population have been documented, with women revealing greater rates of obesity than men (1). Interestingly, mouse models of obesity also reveal sex differences, however in C57Bl/6 mice, male mice typically reach an obese state more frequently and more rapidly than female mice (19–23). We investigated this phenomenon and whether sex differences in the fecal microbiome, as an indicator of the gut microbiome, contribute to the observed sex differences in body weight gain. Therefore, we evaluated the fecal microbiome for male and female mice, fed a low fat or high fat diet. To determine the role of the gut microbiome, we administered an antibiotic cocktail in order to deplete gut microbiome diversity and then evaluated changes to outcome measures. In addition to body weight, we characterized aspects of the lipid profile, including: plasma leptin, plasma adiponectin, plasma free fatty acid profiles, and we evaluated fecal SCFA content. We determined sex differences in body weight despite administration of an antibiotic cocktail. We also determined sex differences in the fecal microbiome due to diet as well as antibiotics. For example, Shannon diversity for Female HF Antibiotics was significantly higher than Male HF Antibiotics. Overall, antibiotics depleted SCFA production. Female HF revealed higher levels of acetic acid, caproic acid, and heptanoic acid compared to Male HF. We observed a number of changes to free fatty acids due to sex, diet, and antibiotics, as well as an interaction effect. We conclude that sex differences in the gut microbiome may contribute to sex differences in obesity, but they do not explain all of the differences. In fact, males and females respond differently to the high fat diet as well as the antibiotic cocktail; further investigation of metabolic differences and response to antibiotics are needed.

## Methods

### Animals, Diets, and Treatments

Sixty-four C57B1/6 mice (The Jackson Laboratory, Bar Harbor, ME, USA) were housed 2–4 to a cage under a controlled, 12-hour light/12-hour dark cycle with *ad libitum* access to food and water. Mice were received at 6 weeks and were allowed to acclimate to the vivarium for 2 weeks. Two-month-old male (n = 32) and female (n = 32) mice were randomly assigned to the following diet groups: High Fat (HF) (n = 31) and Low Fat (LF) (n = 33). Mice were then further divided into antibiotic groups: antibiotic drinking water (n = 36) and normal drinking water (n = 28). This created a total of eight experimental groups: Male LF (n = 8), Male LF Antibiotics (n = 9), Male HF (n = 6), Male HF Antibiotics (n = 9), Female LF (n = 7), Female LF Antibiotics (n = 9), Female HF (n = 7), and Female HF Antibiotics (n = 9).

The LF control diet consisted of (by calorie): 20% protein, 70% carbohydrate, and 10% fat (D12450 K; Research Diets Inc. New Brunswick, NJ, USA). The HF treatment diet consisted of (by calorie): 20% protein, 35% carbohydrate, and 45% fat (D12451; Research Diets Inc., New Brunswick, NJ, USA). Soybean oil and lard were the primary sources of fat for both of these diets, and both diets had the same amount of vitamin and mineral content. The antibiotic cocktail was administered through the drinking water and consisted of 0.5g/L of vancomycin, 1.0 g/L ampicillin, and 1.0 g/L neomycin (Cayman Chemical, Ann Arbor, MI, USA). The diets (HF or LF) were administered for 17 weeks. Antibiotics were added to the drinking water (only for half of the mice) during the last 6 weeks of the total 17-week study. Throughout the 17-week study, body weight was evaluated weekly. Animal protocols were approved by the Furman University Animal Care and Use Committee and carried out according to the guidelines from the National Institute of Health.

### Plasma Collection and Analyses

Mice were anesthetized deeply with isoflurane gas and then decapitated. Trunk blood was collected for plasma analyses at the time of euthanization. Leptin plasma analysis was conducted using a Mouse Leptin ELISA Kit (Crystal Chem, Elk Grove Village, IL, USA; Catalog Number: 90080). Adiponectin plasma analysis was conducted using a Mouse Adiponectin ELISA Kit (Crystal Chem, Elk Grove Village, IL, USA; Catalog Number: 80569). For both ELISAs, the absorbance at A450 and A630 was measured using a BioTek EPOCH 2 microplate reader, with the A630 value subtracted from the A450 value to give the final value used to determine concentration.

Free fatty acid (FFA) analysis was performed by the Medical University of South Carolina Lipidomics Core Facility using high performance liquid chromatography-tandem mass spectrometry (HPLC-MS/MS) on a Thermo Scientific TSQ Quantum Access Max Triple Quadrupole Mass Spectrometer with Thermo Scientific Vanquish uHPLC Chromatography System (Ultra-High Performance Liquid Chromatography). FFA molecular species C12- to C26-saturated and monounsaturated species including Arachidonic acid C20:4, EPA C20:5, and DHA C22:6 were evaluated.

### Microbiome Analyses

Fecal samples for diversity and short chain fatty acid (SCFA) analyses were collected using sterile technique on the last day of the study, in two separate microcentrifuge tubes. For diversity analyses, genomic DNA was isolated from fecal samples using the PowerSoil^®^ DNA Isolation Kit (Qiagen) following the manufacturer’s instructions. As an alternative to the recommended 250mg of soil, approximately 200mg of fecal sample was added to the PowerBeads tube to undergo cell lysis. The purified DNA was eluted from the spin filter using 50uL of solution C6 and stored at −20°C until PCR amplification. The 16S universal Eubacterial primers 515F GTGCCAGCMGCCGCGGTAA and 806R GGACTACVSGGGTATCTAAT were utilized to evaluate the microbial ecology of each sample on the HiSeq 2500 with methods via the bTEFAP^®^ DNA analysis service. Each sample underwent a single-step 30 cycle PCR using HotStarTaq Plus Master Mix Kit (Qiagen, Valencia, CA, USA) and used under the following conditions: 94°C for 3 minutes, followed by 30 cycles of 94°C for 30 seconds; 53°C for 40 seconds and 72°C for 1 minute; after which a final elongation step at 72°C for 5 minutes was performed. Following PCR, all amplicon products from different samples were mixed in equal concentrations and purified using Agencourt Ampure beads (Agencourt Bioscience Corporation, MA, USA). Samples were sequenced utilizing the Illumina MiSeq chemistry following manufacturer’s protocols.

The Q25 sequence data derived from the sequencing process was processed using a proprietary analysis pipeline (www.mrdnalab.com, MR DNA, Shallowater, TX). Briefly, sequences were depleted of barcodes and primers, then short sequences < 200bp were removed, sequences with ambiguous base calls removed, and sequences with homopolymer runs exceeding 6bp were removed. Sequences were then denoised and chimeras removed. Operational taxonomic units were defined after removal of singleton sequences, and clustering at 3% divergence (97% similarity). OTUs were then taxonomically classified using BLASTn against a curated NCBI database and compiled into each taxonomic level, as done previously (21, 22, 24–27).

Short chain fatty acid (SCFA) analysis was conducted by the Duke Proteomics and Metabolomics Core Facility. 50–100 mg of the fecal sample from each mouse was analyzed. Samples were placed in bead blaster CK-14 homogenization tubes (Bertin Corp) and then homogenized on the Precellys 24 bead blaster (Bertin Instruments) at 4°C for 3 cycles of 10 seconds each at 10,000 rpm with a 60-second pause between each burst. The sample extracts were centrifuged at 15,000 rcf for 15 minutes at 4°C. Data collection was performed using LC-MS/MS on a Waters Xevo TQ-S mass spectrometer, including calibration curves for each analyte and a ^13^C_6_ internal standard for each compound (via ^13^C_6_ NPH derivatization reagent). Data analysis was done using Skyline software (www.skyline.ms), and concentrations initially reported in μM. Using the mass of feces homogenized and extracted, and the volume of solvent added, the concentration was then converted to nmol/mg. The SCFA method quantifies 12 SCFAs that can be present in biological samples: acetic acid, propionic acid, iso-butyric acid, butyric acid, 2-methyl butyric acid, iso-valeric acid, valeric acid, 3-methyl valeric acid, iso-caproic acid, caproic acid, heptanoic acid, and octanoic acid. This method is based on validated work published by Han et al. (28).

### Statistical Analysis

All statistical tests were conducted using GraphPad Prism (GraphPad Software, La Jolla, California, USA, www.graphpad.com). Final body weights were analyzed using a two-way ANOVA (sex x treatment) and weekly body weights were analyzed using a repeated measures two-way ANOVA with the Geisser-Greenhouse corrrection. Leptin, adiponectin, FFA, and SCFA levels were analyzed using a two-way ANOVA (sex x treatment). A Tukey’s posthoc analysis was conducted for any measures that indicated statistically significant differences for the ANOVA. Microbiome results were analyzed using a Kruskall-Wallis test followed by a two-stage linear step-up procedure of Benjamini, Krieger, and Yekutieli in order to control for multiple comparisons by controlling the false discovery rate.

## Results

**Figure 1.**
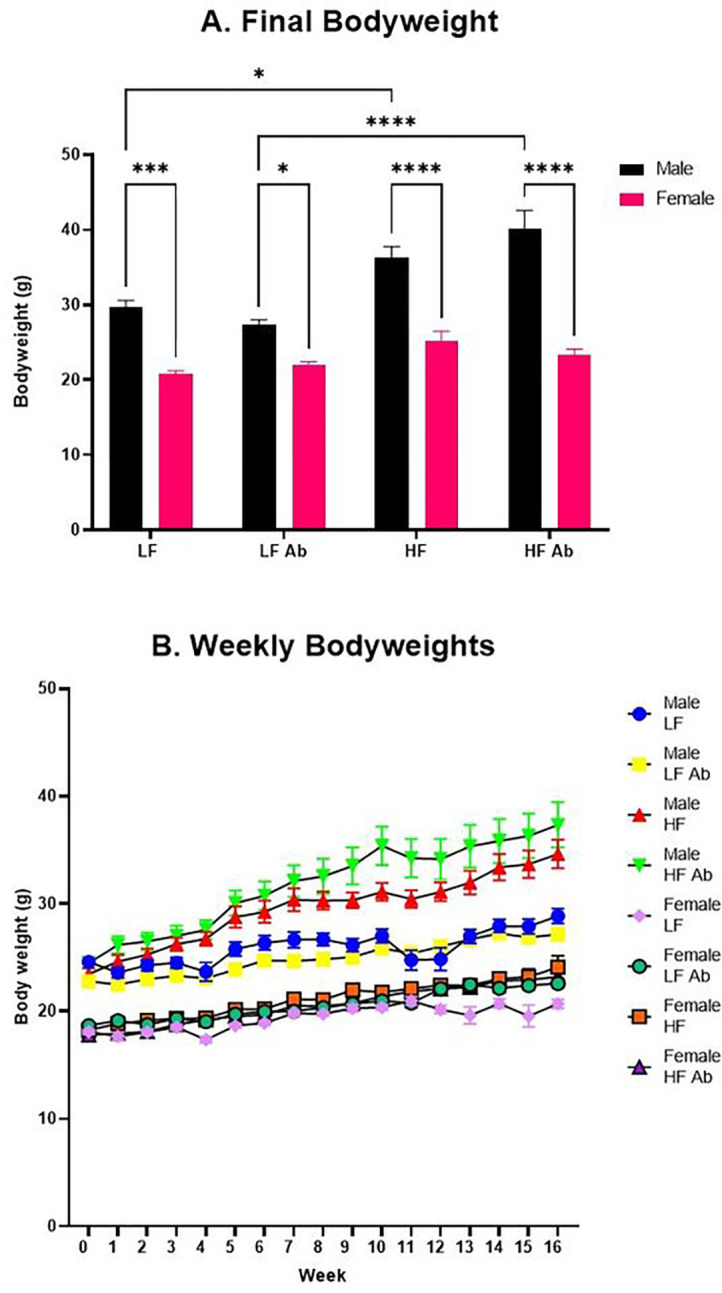
Final Bodyweight and Weekly Bodyweights. (A) Final bodyweights were measured on the day of euthanization after 17 weeks of dietary treatment. (B) Bodyweights were measured weekly throughout the study. Male HF Antibiotics were significantly heavier than all Female groups and Male LF Antibiotics. Male HF were significantly heavier than all Female groups and Male LF.

Final bodyweights were recorded following the conclusion of the 17-week dietary study, on the day of euthanization when plasma and fecal samples were collected. A significant effect of sex (F(1, 56) = 138.7; p < 0.0001), a significant effect of treatment (F(3, 56) = 17.52; p < 0.0001), and a significant interaction effect for sex and treatment (F(3, 56) = 8.068; p = 0.0001) were observed for final bodyweights (Fig. 1A). Male LF revealed significantly greater final bodyweights than Female LF (p = 0.0003), and Male LF Antibiotics had significantly greater final bodyweights than Female LF Antibiotics (p = 0.0397). Male HF had significantly greater final bodyweights than Female HF (p < 0.0001), and Male HF Antibiotics had significantly greater final bodyweights than Female HF Antibiotics (p < 0.0001). Furthermore, Male HF Antibiotics had significantly greater final bodyweights than Male LF Antibiotics (p < 0.0001), and Male HF had significantly greater bodyweights than Male LF (p = 0.0216). No significant differences in final bodyweight were found between diet treatments for females (p = 0.3118), and no significant differences in final bodyweights were found between Antibiotic and non-Antibiotic female groups (LF: p = 0.9978, HF: p = 0.9711).

Bodyweights were measured manually and recorded weekly. A significant effect of treatment (F(7, 56) = 75.54; p < 0.0001), a significant effect of time (F(6.570, 367.9) = 83.39; p < 0.0001), and a significant interaction effect for treatment and time (F(112, 896) = 3.731; p < 0.0001) were observed for weekly bodyweights (Fig. 1B).

### Gut Microbiome Composition and Diversity

After stringent quality sequence curation, a total of 1,680,000 sequences were parsed and 1,610,253 were successfully mapped to zOTUs. 1,605,975 sequences were identified within the Bacteria and Archaea domains and were utilized for final microbiota analyses. The average reads per sample were 33,457. For alpha and beta diversity analysis, samples were rarefied to 20,000 sequences.

**Figure 2.**
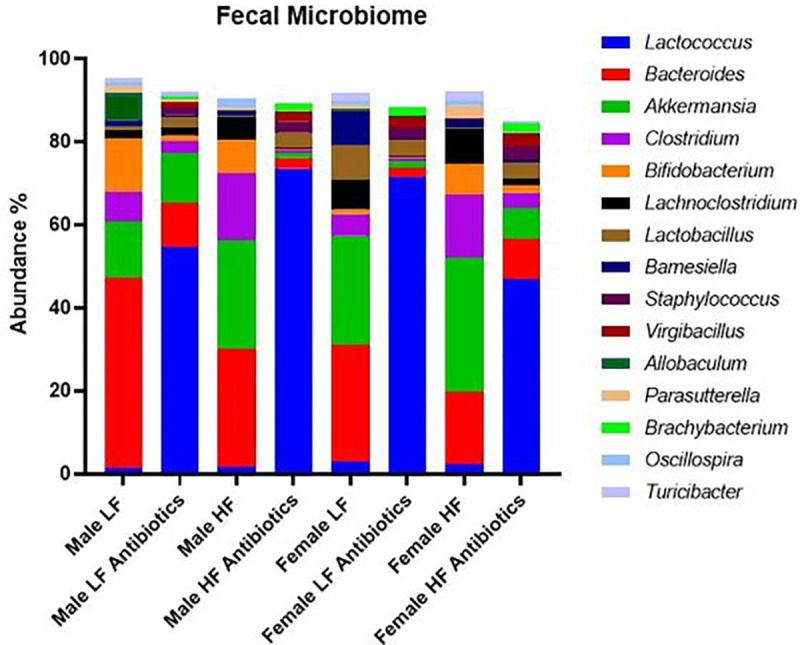
Fecal Microbiome Abundances. Selected genera of bacteria are displayed and compared for average percentage of abundance per group. Antibiotic-treated groups revealed higher percentage of *Lactococcus* compared to non-antibiotic-treated groups. Differences in abundance due to sex, diet, and treatment are revealed.

Antibiotic treatment impacted microbiota diversity as well as specific genus abundances (Fig. 2). For example, *Lactococcus* revealed a significantly higher abundance for antibiotic-treated groups compared to non-antibiotic-treated groups (Relative abundances: Male HF Antibiotics = 73.49%, Female LF Antibiotics = 71.38%, Male LF Antibiotics = 54.51%, and Female HF Antibiotics = 46.83% compared to Female LF = 2.90%, Female HF = 2.23%, Male HF = 1.61%, Male LF = 1.32%). On the other hand, *Bacteroides* abundances were significantly lower for antibiotic-treated groups. However, Female HF (relative abundance of *Bacteroides* = 17.39%) was also low and not significantly different compared to the antibiotic treatment groups (Male LF Antibiotics = 10.82%, Female HF Antibiotics = 9.68%, Male HF Antibiotics = 2.59%, and Female LF Antibiotics = 2.37% compared to Male LF = 45.75%, Male HF = 28.52%, and Female LF = 28.25%). A number of genera of bacteria were lower in abundance for antibiotic-treated groups compared to non-antibiotic-treated groups, including: *Akkermansia, Clostridium, Bifidobacterium, Lachnoclostridium, Oscillospira, Turicibacter, Pseudoflavonifractor.*

We also observed effects on microbiome diversity and abundances due to sex and diet. For example, *Akkermansia* was highest for Female HF (32.5%) as compared to Female LF (26.19%, n.s.), Male LF (13.69%, p = 0.012), and Male HF (26.13%, n.s.). *Barnesiella* was highest for Female LF (8.28%) compared to Female HF (1.88%, p = 0.014), Male LF (1.26%, p = 0.005), and Male HF (0.947%, p = 0.003). *Allobaculum* was significantly higher (p < 0.0001) for Male LF (6.61%) compared to all other groups; Female LF = 0.44%, Female HF = 0.06% and Male HF = 0.09%. Male HF revealed significantly higher (p < 0.0001) abundance of *Peptococcus* (3.93%) compared to all other groups; Male LF = 1.05%, Female LF = 0.03%, Female HF = 0.06%. Female LF revealed significantly higher (p < 0.0001) abundance of *Citrobacter* (2%) compared to all other groups; Male LF = 0.59%, Female HF = 0.26%, and Male HF = 0.04%.

**Figure 3.**
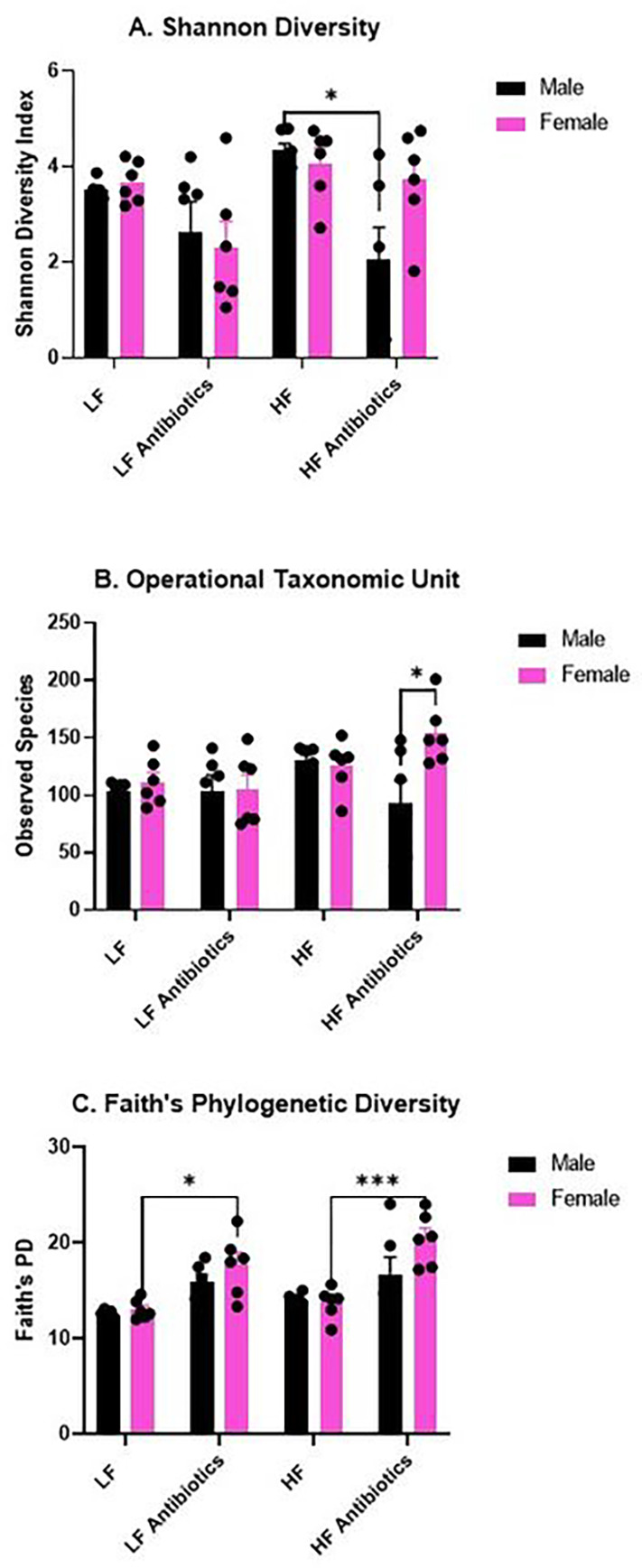
Fecal Microbiome Diversity. (A) Shannon Diversity analysis reveals significantly higher diversity for Male HF compared to Male HF Antibiotics. Interestingly, Shannon Diversity was not significantly altered for Females given antibiotics. (B) Operational Taxonomic Unit counts reveal significantly greater OTUs for Female HF Antibiotics compared to Male High Fat Antibiotics. (C) Faith’s Phylogenetic Diversity analysis reveals significantly higher Faith’s PD for Female HF Antibiotics compared to Female HF as well as Female LF Antibiotics compared to Female LF.

In comparison with results from our previous study (21), which showed a significant effect of antibiotics on the gut microbiome, our current study shows a less significant depletion of gut microbiome diversity from antibiotic treatment (Fig. 3). In the current study, we did not include metronidazole in the antibiotic cocktail due to its ability to cross the blood-brain-barrier (BBB) and other future studies to evaluate neuroinflammation. As in the previous study, the antibiotic cocktail did include the same concentrations of ampicillin, vancomycin, and neomycin. Several genera of bacteria such as *Atopostipes, Brevibacterium, Nosocomiicoccus, Pseudogracilibacillus, Jeotgalicoccus, Corynebacterium, Nocardiopsis, and Salinicoccus* show a significant difference in relative abundance between Antibiotic and non-Antibiotic treatment groups, with the former having a higher relative abundance. None of these genera were analyzed as predominant genera in our previous study (21).

We determined a significant effect of treatment on the Shannon Diversity index (F(3, 40) = 6.192, p = 0.0015; Fig. 3A). Specifically, Male HF revealed a significantly greater Shannon Diversity index compared to Male HF Antibiotics (p = 0.0143). We determined a significant effect of sex on the observed taxonomic units (OTUs; F(1, 40) = 4.251, p = 0.0458) and a significant interaction effect of sex and treatment (F(3, 40) = 3.397, p = 0.0269). Specifically, Male HF Antibiotics revealed significantly lower OTUs compared to Female HF Antibiotics (p = 0.0119; Fig. 3B). Last, we determined a significant effect of treatment (F(3, 40) = 15.69, p < 0.0001) and a significant effect of sex (F(1, 40) = 4.575, p = 0.0386) on Faith’s phylogenetic diversity (Fig. 3C). Specifically, Female LF Antibiotics displayed a significantly higher Faith’s PD compared to Female LF (p = 0.0274) and Female HF Antibiotics displayed a significantly higher Faith’s PD compared to Female HF (p = 0.0004). Female HF Antibiotics also revealed a significantly higher Faith’s PD compared to Male HF (p = 0.0006).

### Short Chain Fatty Acid Analysis

**Figure 4.**
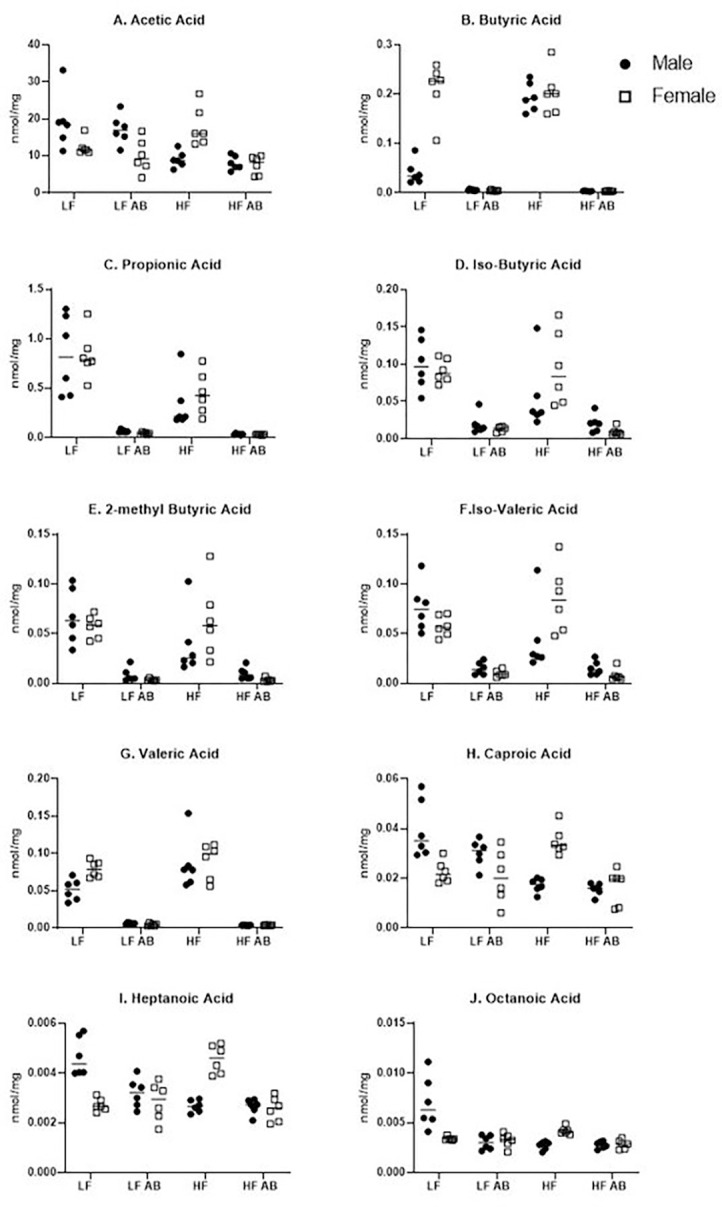
Short Chain Fatty Acid Analysis. Ten SCFAs were measured in fecal samples from each group.

Ten short chain fatty acids (SCFAs) were analyzed in the fecal samples that were collected at the conclusion of the 17-week study (Fig. 4); two SCFAs were below the level of detection in these samples: 3-methyl valeric acid and iso-caproic acid. All ten of the measurable SCFAs revealed a significant effect of treatment: Acetic acid (F(3, 40) = 8.250, p = 0.0002; Fig. 4A), Butyric acid (F(3, 40) = 137.2, p < 0.0001; Fig. 4B), Propionic acid (F(3, 40) = 40.56, p < 0.0001; Fig. 4C), Iso-Butyric Acid (F(3, 40) = 24.93, p < 0.0001; Fig. 4D, 2-methyl Butyric Acid (F(3, 40) = 23.96, p < 0.0001; Fig. 4E), Iso-Valeric Acid (F(3, 40) = 28.66, p < 0.0001; Fig. 4F), Valeric Acid (F(3, 40) = 82.37, p < 0.0001; Fig. 4G), Caproic Acid (F(3, 40) = 9.494, p < 0.0001; Fig. 4H), Heptanoic Acid (F(3, 40) = 10.88, p < 0.0001; Fig. 4I), and Octanoic Acid (F(3, 40) = 13.03, p < 0.0001; Fig. 4J). Acetic acid, butyric acid, iso-valeric acid, caproic acid, heptanoic acid, and octanoic acid also revealed an interaction (sex x treatment) effect: Acetic acid (F(3, 40) = 9.831, p < 0.0001), Butyric acid (F(3, 40) = 25.82, p < 0.0001), Iso-valeric acid (F(3, 40) = 5.312, p = 0.0035), Caproic acid (F(3, 40) = 14.03, p < 0.0001, Heptanoic acid (F(3, 40) = 25.33, p < 0.0001), and Octanoic acid (F(3, 40) = 13.56, p < 0.0001). Lastly, butyric acid also revealed an effect of sex (F(1, 40) = 28.93, p < 0.0001).

### Plasma Leptin and Adiponectin Concentrations

**Figure 5.**
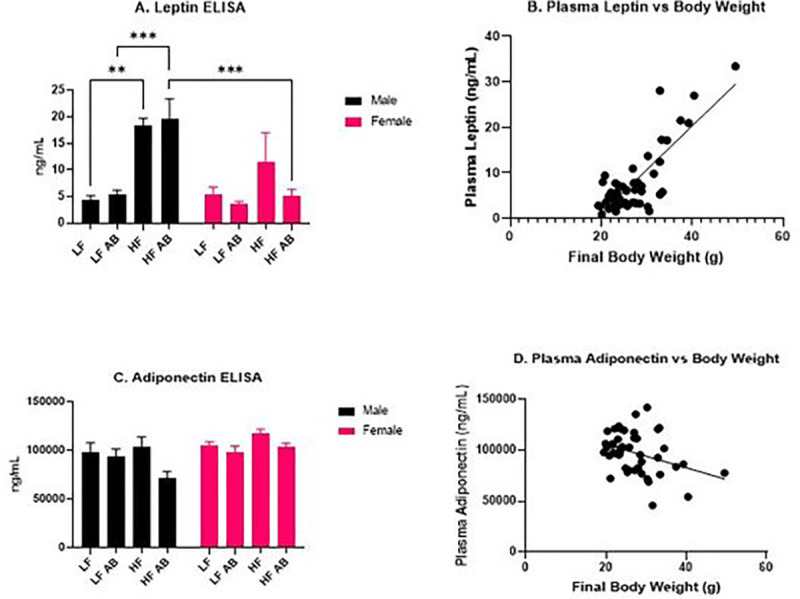
Plasma Leptin and Adiponectin Analysis. (A) Plasma leptin levels. (B) Positive correlation between plasma leptin levels and bodyweight. (C) Plasma adiponectin levels. (D) Negative correlation between plasma adiponectin levels and bodyweight.

Plasma leptin and adiponectin concentrations were determined following the conclusion of the 17-week study. A significant effect of sex (F(1, 39) = 13.28, p = 0.0008), a significant effect of treatment (F(3, 39) = 11.36, p < 0.0001), and a significant interaction effect for sex and treatment (F(3, 39) = 5.719, p = 0.0024) were observed in plasma leptin concentrations (Fig. 5A). Male HF had significantly higher plasma leptin concentrations than Male LF (p = 0.0046), which was expected given the significantly higher body weight for Male HF compared to Male LF. Male HF Antibiotics had significantly higher plasma leptin concentrations compared to Male LF Antibiotics (p = 0.0002). Male HF Antibiotics had significantly higher plasma leptin concentrations than Female HF Antibiotics (p = 0.0002), supporting the significant effect of sex on plasma leptin concentration, which was also expected given the sex differences in body weight. A significant positive correlation (r = 0.6780, p < 0.0001) was found between the final bodyweight and plasma leptin concentration, demonstrating that mice with higher bodyweights had a higher concentration of plasma leptin (Fig. 5B).

A significant effect of sex (F(1, 38) = 7.017, p = 0.0117) and a significant effect of treatment (F(3, 38) = 2.909, p = 0.0469) were determined for plasma adiponectin levels (Fig. 5C). And, plasma adiponectin negatively correlated with body weight (r = −0.3517, p = 0.0165; Fig. 5D).

### Free Fatty Acid Analysis

Free fatty acid concentrations in the plasma were measured following the conclusion of the 17-week study (Table 1). For C16:0, a significant interaction effect of sex and treatment was observed (F(3, 40) = 12.24, p < 0.0001). For C16:1, a significant effect of treatment was observed (F(3, 40) = 5.564, p = 0.0027). For C18:0, a significant interaction effect of sex and treatment was observed (F(3, 40) = 11.26, p < 0.0001). For C20:1, a significant effect of sex (F(1, 40) = 14.78, p = 0.0004) and a significant interaction effect of sex and treatment (F(3, 40) = 6.911, p = 0.0007) were observed. For C20:4, a significant effect of treatment (F(3, 40) = 12.48, p < 0.0001) and a significant interaction effect of sex and treatment (F(3, 40) = 7.273, p = 0.0005) were observed. For C22:0, a significant effect of treatment (F(3, 40) = 5.480, p = 0.003) and a significant interaction effect of sex and treatment (F(3, 40) = 6.736, p = 0.0009) were observed. For C22:1, a significant effect of treatment (F(3, 40) = 2.880, p = 0.0477) and a significant effect of sex (F(1, 40) = 42.45, p < 0.0001) were observed. For C24:0, a significant interaction effect of sex and treatment (F(3, 40) = 3.167, p = 0.0347) was observed. For C24:1, a significant interaction effect of sex and treatment (F(3, 40) = 2.874, p = 0.048) was observed. For C26:0, a significant interaction effect of sex and treatment (F(3, 40) = 3.922, p = 0.0152) was observed. No significant effect of sex, treatment, or interaction effect of sex and treatment was observed for C14:0, C18:1, or C20:0.

## Discussion

To gain more information about sex differences in obesity, we characterized and compared multiple aspects of the lipid profile as well as the fecal microbiome between male and female mice. In C57Bl/6 mice, male mice typically gain more weight, more rapidly, compared to female mice (19–23). We observed this effect in our current study; Male HF revealed significantly greater bodyweights than Female HF (p = 0.0479). This indicates a resistance to diet-induced obesity in female mice as compared to male mice, in accordance with previous studies. Prior research with germ-free (GF) mice also noted a resistance to diet-induced obesity in both sexes due to an increased metabolism of fatty acids (29). We hypothesized a role of the gut microbiome for sex differences in obesity.

Interestingly, statistics gathered on obesity prevalence in the human population indicate a higher rate of severe obesity (BMI ≥ 40) for females compared to males. In 2018, the NHANES study revealed a higher prevalence of severe obesity in women (11.5%) compared to men (6.9%), which was highest for ages 40–59. However, there was no significant difference for age-adjusted obesity (BMI ≥ 30) prevalence between men and women at this time. But, for non-Hispanic black women, the obesity prevalence was 56.9% compared to 41.1% non-Hispanic black males, and 39.8% for non-Hispanic white women compared to 44.7% non-Hispanic white males. There were no major differences between non-Hispanic Asian women (17.2%) and non-Hispanic Asian men (17.5%) or Hispanic women (43.7%) and Hispanic men (45.7%) (30). Data for worldwide obesity indicate 11% of men and 15% of women were obese in 2016 (World Health Organization, 2018), indicating sex differences in obesity for the human population and a need for interventions and prevention that takes sex into account. Women tend to have more subcutaneous fat whereas men have more visceral fat; increased visceral fat puts men at higher risk for cardiovascular disease. The distribution of fat in subcutaneous stores for women is attributed to sex-hormone signaling in adipocytes. However, when hormone changes occur during menopause, body fat is re-distributed to visceral stores and the risk of cardiovascular disease rises for women (32). More research is necessary to understand why mouse models of obesity reveal increased adiposity for male mice versus female mice whereas the human population reveals the opposite. Hormone status is an important factor as severe obesity rates are highest for post-menopausal women (33, 34) and most animal studies utilize young, sexually naïve rodents (19–23, 35). Previous work has shown that pre-menopausal women have slower transit time through the gastrointestinal tract compared to men, and ovariectomized female rats have increased transit time that is rescued with administration of estrogen, indicating an important role for hormones in the gastrointestinal tract and a factor influencing microbiome composition. With longer transit time, food particles interact with the intestines for a longer time and affect fermentation, breakdown, and production of metabolites (36).

A role for the gut microbiome in obesity has been proposed (4, 6, 37–39). The gut microbiota contribute to digestion, metabolism, and nutrient absorption (3). To examine the contribution of gut microbiome diversity to sex differences in obesity, we depleted the gut microbiome using an antibiotic cocktail for six weeks at the end of the study. Previous research reported a significant depletion of gut microbiome diversity from antibiotic treatment when utilizing an antibiotic cocktail of metronidazole, ampicillin, neomycin, and vancomycin (21). We hypothesized that similar effects would be observed for depleting gut microbiome diversity while using a cocktail of just ampicillin, neomycin, and vancomycin given the utilization of this antibiotic cocktail in other studies (40, 41). Our findings suggest the removal of metronidazole from the antibiotic cocktail led to decreased effectiveness of the treatment in significantly reducing gut microbiome diversity. Predominant genera measured following administration of the antibiotic cocktail used in this study differ from those present with the previously mentioned antibiotic cocktail containing metronidazole. Due to findings that implicate the gut microbiome as a contributor to sex differences in obesity (10, 12, 21, 42, 43), we hypothesized that depleting bacteria present in the gut microbiome could lead to a normalization of body weights. However, we did not observe this effect during the time frame of the study. Instead, sex differences in bodyweight persisted, with Male HF Antibiotics gaining even more weight compared to Male HF (not statistically significant) whereas Female HF Antibiotics did not and remained significantly less bodyweight compared to males.

A number of human and animal studies have investigated specific bacteria that impact body weight, adiposity, and metabolism. For example, obese subjects tend to have a higher *Firmicutes* to *Bacteroidetes* ratio. Bacteroides thetaiotaomicron and *Akkermansia muciniphila* have been linked with lower adiposity whereas Acinetobacter, *Blautia*, and *Dorea* have been linked with increased adiposity (44). In our study, we determined the highest abundance of *Akkermansia* for Female HF, which could explain some of the resistance to obesity for this group. We also determined high levels of *Akkermansia* for Female LF, which could be an inherent sex difference contributing to these sex differences in adiposity. We also determined the highest level of *Dorea* for Male HF, supporting the increased adiposity for this group compared to all other groups. Interestingly though, *Dorea* was significantly lower for Male HF Antibiotics (p < 0.0001) and therefore it cannot fully explain differences in adiposity given that Male HF Antibiotics had the highest adiposity of all groups. Mice administered antibiotics did have growth of *Acinetobacter*, with Female HF Antibiotics revealing the highest levels, and all groups that received normal drinking water did not have *Acinetobacter* present in their fecal samples.

Proposed mechanisms for the role of the gut microbiome include changes to nutrient metabolism as well the impact of SCFA production on metabolism. SCFAs are produced by bacterial fermentation of non-digestible carbohydrates, and they can regulate satiety, cell differentiation, cell apoptosis, and colon motility (45). Previous work has shown higher butyrate, acetate, and propionate in feces for obese individuals (46, 47). The proposed mechanism for this change during obesity is a decrease in SCFA-producing bacteria for obese individuals, decreased absorption of SCFA, and then increased SCFA excretion in feces (48). In our study, we observed increased fecal acetic acid for Female HF compared to Male HF, indicating a sex difference. Male HF had significantly greater acetic acid than Male LF, but Female HF values were not significantly different from Female LF, indicating a sex x treatment interaction effect. Fecal butyric acid was high for both Female LF and Female HF, whereas males revealed a diet treatment effect with significantly higher butyric acid after the HF diet. Propionic acid did not reveal a sex difference, with both sexes and dietary treatments revealing similar levels. Female HF also had increased fecal iso-butyric acid, 2-methyl butyric acid, iso-valeric acid, caproic acid, and heptanoic acid compared to Male HF. Given previous conclusions that increased fecal SCFA levels are associated with obesity due to decreased SCFA production and absorption, these data for higher fecal SCFA in females on the high fat diet are surprising given that females did not become obese like male mice on the high fat diet. However, sex differences in SCFA production are not yet well-characterized. More research is needed to discern sex differences in SCFA production, metabolism, and absorption as an important factor in obesity.

We determined a depletion of SCFA production for butyric acid, propionic acid, iso-butyric acid, 2-methyl butyric acid, iso-valeric acid, and valeric acid following antibiotic treatment. Interestingly, other SCFAs were not as affected by antibiotic treatment: acetic acid, caproic acid, heptanoic acid, and octanoic acid. There were no significant sex differences in SCFAs following antibiotic treatment. But, we did determine sex differences in fecal microbiome genera abundances due to diet as well as antibiotic treatment. *Bacteroides* spp., *Bifidobacterium* spp., *Prevotella* spp., *Ruminococcus* spp., *Clostridium* spp., and *Streptococcus* spp. are major producers of acetate via an acetyl-coA hydrolase (49). *Bacteroides* was significantly reduced for all antibiotic-treated groups, and it was also lower for Female LF compared to Male LF (p = 0.049) and Female HF compared to Male HF (not statistically significant), supporting a sex difference and sex x treatment interaction effect. *Bifidobacterium* was not significantly reduced by antibiotic treatment for all groups. Only Male HF Antibiotics revealed statistically significant reduced levels compared to Male HF (p = 0.024) and Male LF had the highest amount of *Bifidobacterium*. On the other hand, Male HF had the highest amount of *Ruminococcus* and Male LF had the lowest (p = 0.001). Antibiotic treatment did not significantly deplete *Ruminoccocus* for any of the groups except for Male HF compared to Male HF Antibiotics (p = 0.01). These variable changes in enteric bacteria could explain the lack of a significant depletion in acetic acid levels for our study. Butyrate can be produced by a number of species with *Ruminococcus bromii*, some *Coprococcus* species, *Faecalibacterium prausnitzii, Eubacterium rectale, Eubacterium hallii, and Ruminococcus bromii* as the major producers in human metabolism (49). We determined the highest abundance of *Eubacterium rectale* for Female HF, Male HF, and Female LF, supporting higher levels of butyrate production for these groups. We also determined a reduction in *Eubacterium rectale* for Male HF Antibiotics compared to Male HF and Female LF Antibiotics compared to Female LF, supporting one of the contributors to significantly deplete butyrate production for antibiotic-treated mice. Interestingly, *Coprococcus spp*. was highest in abundance for Female HF and Female HF Antibiotics compared to the other groups (Female HF was significantly greater than Male HF (p = 0.0208), Male LF (p = 0.0208), and Female LF Antibiotics (p = 0.0208) because these three groups revealed no *Coprococcus spp*.). We also did not find any *Ruminococcus bromii* for any of the groups (with the exception of very low levels for one Male HF Antibiotics mouse and one Male LF Antibiotics mouse). Propionate can be produced by a few different species and biochemical pathways, with *Akkermansia municiphilla* as the major propionate producer (49). We determined Female HF to have the highest abundance of *Akkermansia municiphilla* and a significant reduction in abundance for Male HF Antibiotics compared to Male HF (p = 0.0035) and Female LF Antibiotics compared to Female LF (p = 0.0138), providing some support to the depletion of propionic acid in fecal samples from antibiotic-treated mice in our study. Overall, there are a number of bacterial species contributing to the observed sex differences, dietary treatment effects, and antibiotic treatment effects; it is important to consider the full microbiota landscape rather than individual species for these effects. Furthermore, more research is necessary to better understand the interactions between various microbiota and their effects on SCFA production as well as metabolism as a whole.

Plasma FFA levels are typically elevated in obesity due to increased release from adipose tissue and reduced clearance, this can also lead to insulin resistance as well as other aspects of metabolic syndrome (50). Interestingly, Male HF did not reveal significantly higher FFA levels compared to Male LF, and Female HF did not reveal significantly higher FFA levels compared to Female LF. Instead, we observed a greater effect on FFA levels due to antibiotic treatment as well as sex differences. Female LF revealed significantly higher levels of palmitic acid (C16:0) compared to Male LF (p = 0.0029). Given that Female LF do not gain very much weight, this elevated circulating palmitic acid may be due to a lack of absorption from the diet. The LF diet does comprise a low amount of calories from fat (10%), however it is formulated with both soybean oil and lard. Interestingly, Female HF had significantly lower levels of plasma palmitic acid than Female LF (p = 0.0012), even though the HF diet contains 45% calories from fat. This may point to differences in lipid absorption, in part due to microbiome differences rather than hormones alone. We also observed significant differences in plasma palmitic acid due to antibiotic treatment, further supporting a role of the microbiome. Male LF Antibiotics had significantly elevated palmitic acid compared to Male LF (p = 0.0104), with comparable values to Female LF whereas Female LF Antibiotics had significantly lower levels of plasma palmitic acid than Female LF (p = 0.0073), comparable to Male LF. Female LF and Female HF Antibiotics also had higher plasma stearic acid (C18:0) compared to all other groups. On the other hand, Male HF Antibiotics revealed the highest level of arachidonic acid (C20:4), a polyunsaturated omega-6 fatty acid, compared to all other groups. Previous work by Seeger and Murphy (51) investigated differences in fatty acid uptake and trafficking into multiple organs between C57Bl/6 mice and Swiss Webster mice and determined significant differences between these strains for heart fatty acid uptake and trafficking, particularly for 20:4n-6. Seeger and Murphy highlight the importance in recognizing strain differences when comparing results for lipid metabolism. These studies were conducted in male mice; it will be important to determine and recognize sex differences in lipid metabolism as well.

Our study confirmed the presence of a statistically significant difference in plasma leptin concentrations between Male HF Antibiotics and Female LF Antibiotics treatment groups (p = 0.0002), with males having higher concentrations of leptin than females. We also confirmed previous findings in which a significant positive correlation exists between bodyweight and plasma leptin concentrations (52). The observed sex differences in plasma leptin were expected given that male mice had significantly higher body weights than female mice in this study. An effect of sex (p = 0.0117) and an effect of treatment (p = 0.0469) were also determined for adiponectin; Male HF Antibiotics had the lowest plasma adiponectin levels while Female HF had the highest.

### Perspectives and Significance

We characterized a number of sex differences due to diet as well as antibiotics treatment. We hypothesized that depletion of gut microbiome diversity could impact body weight and potentially normalize sex differences in diet-induced obesity for this mouse model. We did not observe a normalization of body weight between sexes following administration of antibiotics. There are limitations to our study, including only six weeks of antibiotics treatment after already eleven weeks of dietary treatment, and we know that there can be off-target effects of antibiotics outside of microbiota depletion. However, the findings herein, including characterization of changes to the fecal microbiota, SCFA production, and plasma lipid profile due to sex dietary treatment, and antibiotics point to the importance of better understanding sex differences in response to diet and antibiotics. Understanding sex differences in metabolism is necessary as personalized medicine evolves and new therapeutics for the obesity epidemic are developed.

## Figures and Tables

**Table 1:** Free Fatty Acid Analysis. Statistical significance indicated by superscript letters to indicate groups that are significantly different.

	C14:0	C16:0	C16:1	C18:0	C18:1	C20:0	C20:1	C20:4
**Male LF**	0.008	0.214^a^	0.020^a^	0.138^a^	0.062	0.038	0.002^a,d^	0.099^a^
**Male LF AB**	0.014	0.341^b^	0.049^b^	0.214^a^	0.107	0.035	0.004^b,c^	0.152^a,c^
**Male HF**	0.008	0.281^a^	0.015^a^	0.190^a^	0.088	0.031	0.003^a,c^	0.137^a,c^
**Male HF AB**	0.012	0.274^a^	0.021^a^	0.182^a^	0.112	0.028	0.004^c,d^	0.249^b^
**Female LF**	0.010	0.356^b^	0.023^a^	0.239^b^	0.082	0.022	0.003^a^	0.145^a^-^c^
**Female LF AB**	0.010	0.225^a^	0.026^a^	0.137^a^	0.094	0.038	0.001^a^	0.145^a,c^
**Female HF**	0.009	0.204^a^	0.014^a^	0.137^a^	0.091	0.03	0.001^a^	0.178^c^
**Female HF AB**	0.011	0.313^b^	0.014^a^	0.218^b^	0.086	0.024	0.002^a,c^	0.173^c^

## Highlights

- Characterization of the fecal microbiome at the genus level for male and female C57Bl/6 mice on a low fat diet, high diet, with and without antibiotic treatment
- Characterization of short chain fatty acid production for male and female C57Bl/6 mice on a low fat diet, high diet, with and without antibiotic treatment
- Characterization of circulating free fatty acids for male and female C57Bl/6 mice on a low fat diet, high diet, with and without antibiotic treatment
- Male mice gained the most weight on a high fat diet, following antibiotic treatment, compared to all other groups
- Significant depletion of the microbiome via antibiotic treatment does not decrease body weight and does not normalize sex differences in obesity
